# The global status of genetic counselors in 2023: What has changed in the past 5 years?

**DOI:** 10.1016/j.gimo.2024.101887

**Published:** 2024-08-08

**Authors:** Kelly E. Ormond, Peter James Abad, Rhona MacLeod, Masakazu Nishigaki, Tina-Marié Wessels

**Affiliations:** 1 Health Ethics and Policy Lab, Department of Health Sciences and Technology, ETH-Zurich, Switzerland; 2 Department of Genetics, Stanford School of Medicine, Stanford, CA; 3 University of Iowa College of Nursing, Iowa City, IA; 4 University of the Philippines Manila College of Nursing, Manila, Philippines; 5 Manchester Centre for Genomic Medicine, St Mary’s Hospital, Manchester University NHS Foundation Trust, Manchester, United Kingdom; 6 School of Biological Sciences, Division of Evolution, Infection and Genomics, University of Manchester, Manchester, United Kingdom; 7 Department of Genetic Counseling, International University of Health and Welfare, Tokyo, Japan; 8 Division Human Genetics, University of Cape Town, Cape Town, South Africa

**Keywords:** Genetic counselor, International, Regulation, Training, Workforce

## Abstract

**Purpose:**

The profession of genetic counselors has existed for over 50 years. This article provides an update on the global state of the genetic counseling (GC) profession in 2022 and 2023.

**Methods:**

We used a survey approach to collect data from individuals who were identified as being leaders in GC practice and/or education around the world.

**Results:**

Based on responses provided between October 2023 and January 2024, we estimate that there are over 10,250 genetic counselors in over 45 countries around the world. These numbers have increased significantly in the past 5 years, when there were ∼7000 genetic counselors. Key factors identified as driving the increase in genetic counselors are the number of training programs that have developed (>130 globally, mostly at a master’s degree level) and a growing number of national biobanks and/or population screening programs that require GC as part of the process. There is tremendous variability in how genetic counselors are regulated, with only a few countries holding national statutory regulation processes. There are many commonalities, but the GC profession appears nuanced to their specific country and its wider socio-political context and history.

**Conclusion:**

We hope that genetic counselors internationally will come together to assist each other in all aspects: training, statutory regulation, developing the workforce, and establishing GC as an academic discipline. We envision that establishing an international organization for the profession of genetic counselors might maintain such international connections and provide relevant data in future years.

## Introduction

In 2018, the article “The Global State of the Genetic Counseling Profession” was first published online.^1^ The article was an early attempt to provide a summary of the genetic counseling (GC)^a^ profession across the world. The information was primarily based on presentations at the Transnational Alliance for Genetic Counseling (TAGC) conference in Barcelona in 2016 and information obtained through the TAGC member group, the National Society of Genetic Counselors (NSGC), and the 2017 World Congress of Genetic Counseling. In 2017, it was estimated that there were over 7000 trained genetic counselors practicing in 28 Countries.^1^

In the 5 years since the Abacan et al^1^ publication, there have been many published articles addressing various issues related to the global genetic counseling profession. Although this article does not intend to perform a systematic review of this literature, it does provide some information on recent work. For example, some articles describe the status of the profession in a single country, such as the Philippines, Austria, Korea, or Japan,^2^, ^3^, ^4^, ^5^, ^6^ or historical and current roles of genetic counselors in a single country, for example, in the United Kingdom^7^^,^^8^ or United States.^9^ Others discussed the status and future of genomic medicine more broadly in a specific country or region, such as Egypt,^10^ the Middle East, or Scandinavia.^11^^,^^12^

Some publications were more specific to GC service models and development^13^ in specific countries such as Romania. Many focus on how the COVID-19 pandemic affected telemedicine provision of GC in the United States,^14^, ^15^, ^16^ Italy,^17^ Hong Kong Special Administrative Region (SAR),^18^ Israel,^19^ the Philippines,^20^ Portugal,^21^ South Africa,^22^ Tuscany,^23^ and others. Subsequent to this, evidence-based guidelines for telehealth have been developed.^24^ There are few articles examining how services are delivered across many countries. One such study was by Ormond et al^25^ (2023), which conducted an exploratory survey of 189 genetic counselors from 22 countries and documented specific roles in clinical practice. This study found that there were 20 activities (such as obtaining medical histories, discussing testing options, and performing risk assessment) where >74% of genetic counselors reported that they included the activity in a typical GC session.

Beyond these clinical areas, there are articles that have emphasized the genetic counselors’ role in research,^26^^,^^27^ professional issues of inclusivity and diversity,^28^, ^29^, ^30^ and the ongoing challenge of credentialing.^31^ Few articles described regional updates in genetic counselor training.^32^^,^^33^

Through discussion among members of the TAGC board of directors (BOD), the authors were interested in documenting the current (2022-2023) status of the profession, as well as how the genetic counseling profession has changed over the past 5 years and what issues have arisen during this time. This article aims to provide a comprehensive and current overview of the GC profession around the globe and its evolution since 2017, when data were collected for the Abacan et al^1^ article.

## Materials and Methods

The information presented here was obtained through collecting data from an international online survey, supplemented by review of relevant references and conversations with international GC colleagues. This project did not collect individual-level human data and therefore was exempt from human subjects review.

### Survey

The survey (see Supplemental Methods) was novelly developed and pilot tested by the coauthors, who are genetic counselors and educators internationally. The primary research question was to describe the state of genetic counselors as a profession around the globe. We acknowledge that GC, as defined by NSGC’s Definition Task Force^34^ (2006), is an activity that can be performed by a number of health care professionals, not exclusively by genetic counselors; in fact in many countries it is still an activity performed exclusively by physicians. However, as in the Abacan et al^1^ (2019) article, we refer to the GC profession as allied healthcare providers with specific (often master’s level) training in Medical Genetics and counseling skills. Cohen^35^ provides a more comprehensive depiction and described genetic counselors as “…master’s-level trained individuals who apply their skills to assist patients in understanding the genetic etiology of disease, assessing risk for genetic disease, and providing support to those who are encountering a genetic disorder in themselves or family members.”

The survey, which was provided only in English, consisted of questions that primarily assessed the number and status of genetic counselors at the end of 2022 and the current status of genetic counselor training programs and genetic counselor regulation. Open-ended questions assessed changes over the past 5 years across a range of areas (curriculum, clinical training, research, aspects of clinical practice such as roles and specialty areas, changes in telehealth since COVID-19 pandemic, regulation, billing, and reimbursement), and perceived areas of challenge and opportunity over the upcoming 5 years. The survey specified that the person completing the survey should consult with relevant leaders within their country (for example, a professional organization president) and cite websites and published articles as much as possible to provide justification for the data provided.

### Participants and recruitment

The target audience was any individual who would be able to provide summary information about the state of GC in their country. The authors utilized several strategies to identify this information. First, as was done in the Abacan et al^1^ article, we utilized the BOD of TAGC because these individuals were previously identified as leaders within their country and region within the GC field. Second, the TAGC listserve, with approximately 245 subscribers, was used to identify individuals who had previously expressed interest in global issues around GC and who were themselves, or had contacts, in additional countries that had genetic counselors but were not represented on the TAGC BOD. Third, we utilized a snowball sampling approach, asking members of the TAGC listserve and BOD to share the survey with relevant individuals in countries that were not yet represented. We also asked survey respondents to provide contact information for any additional unrepresented countries.

### Survey distribution

The survey was administered online via Qualtrics, and electronic PDF copies were sent as part of the recruitment email and could also be used for completion. Data were collected between September 2023 and January 2024. To ensure that we interpreted the data accurately, we gave respondents a week to review a draft of the article and to provide additional corrections or comments in January 2024. We received 1 additional result by personal email in April 2024.

For most countries there was a single survey respondent, although multiple responses were received for 2 countries. When discrepant data were received, including information that seemed contradictory to what was reported by Abacan et al,^1^ the participants were emailed for clarification.

### Analysis

The surveys were reviewed individually, and descriptive information is provided in this article. References and relevant websites were reviewed to confirm the accuracy of the data. In the event that we were not able to receive an updated state of the profession for a particular country that was reported in the Abacan et al^1^ article, we conservatively estimated that there were no changes to the numbers of genetic counselors since that time.

For the open-ended comments, a code book was developed by a single author (K.E.O.) and applied by 2 coauthors (R.M. and K.E.O.) to identify key themes, which were then discussed and approved by all coauthors. We present the high-level themes and illustrative quotes in this article.

## Results

Responses, including completed surveys and private emails, were received representing over 45 countries across 6 continents. We list in the acknowledgments section the names of participants who shared information and gave permission for acknowledgment, many of whom held leadership positions in the various GC professional organizations or GC training programs for the country of which they responded.

**Figure 1:**
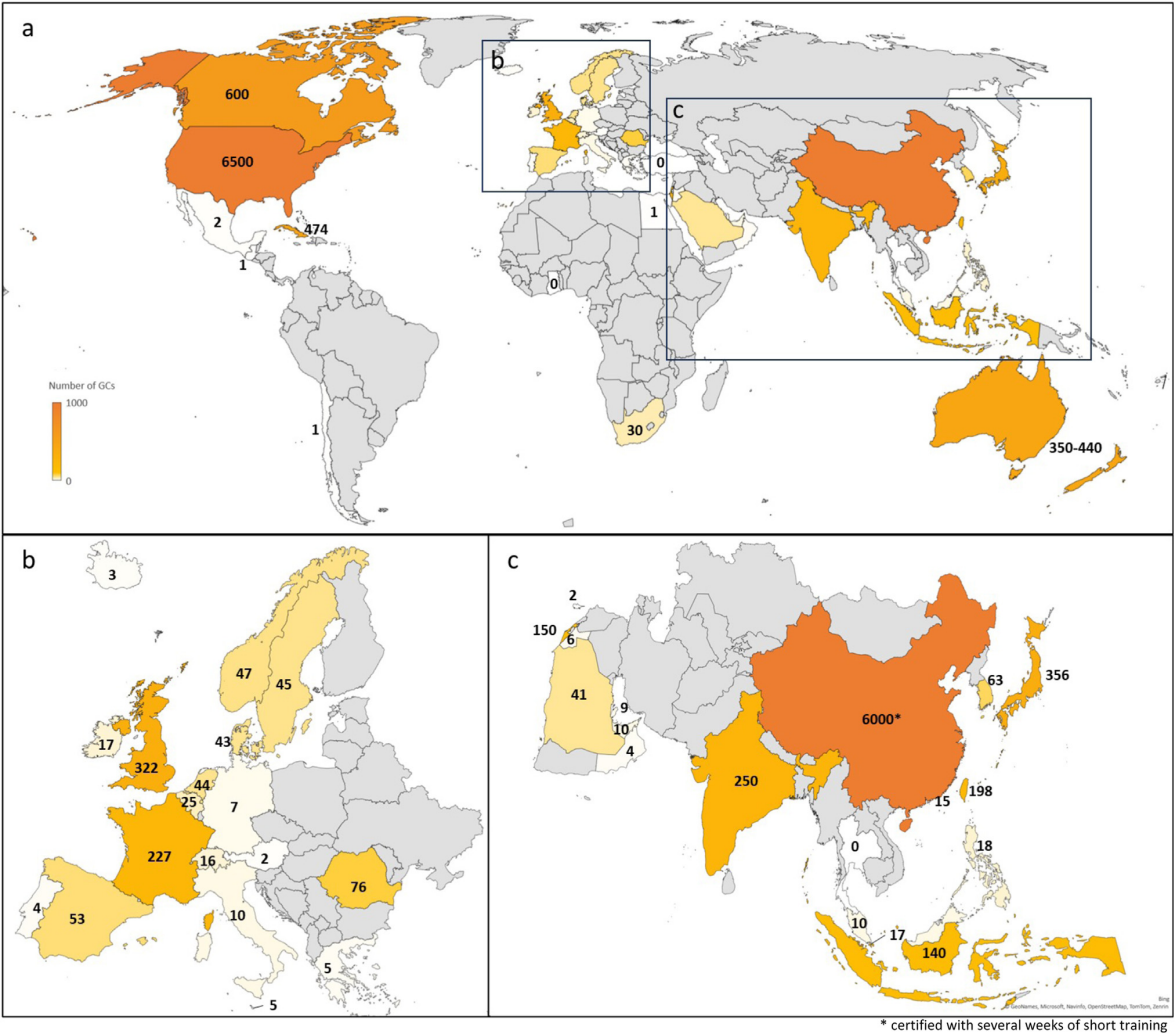
**Distribution of genetic counselors (GCs) over the world**.

Table 1 and Figure 1 summarize the updated global numbers of genetic counselors, which we estimate at greater than 10,250 genetic counselors in 2022. Table 2 summarizes the current status of credentialing, including by professional bodies and statutory regulation. Table 3 presents information regarding relevant professional organizations globally. Below, we will present a summary of major changes and challenges that were reported by the respondents. Short summaries by region are presented as well. We include published articles and website references to document the information sources when feasible.

**Table 1:** Overview of the number, training, and regulation of genetic counselors globally

Country	2022 Population Estimate	Estimated Number of GCs		No. of GC Training Programs		Training of Genetic Counselors						Regulation of Genetic Counselors^a^		
						Year First Training Program Started	No. Under Dev’p	No. Closed (Past 5 y)	Degrees Awarded	Length of Training (y)	No. in Training Per Year	Nat’l Regulation	State or Province Regulation	Professional Body Regulation
		2017	2022	2017^b^	2022									
**North America**														
Canada	40 million	∼350	∼600	5	5	1984	2	0	MSc	2	54	No	No	Yes
United States of America	333.354 million	∼4000	>6500	39	56	1969	6	1	MS, MGC, PhD	2	591	No	Yes (35/50 states)	Yes
**Central and South America**														
Brazil	203 million	nd	nd	1	1	2015	-	-	MGC	-	-	nd	nd	nd
Chile	19.6 million	1	1	-	1	2022	-	-	PGDip	-	-	nd	nd	nd
Cuba	11.086 million	∼900	474	1	nd	1999	nd	nd	MS	nd	50 every other y	No	No	No
Guatemala	17.358 million	nd	1	nd	0	-	-	-	-	-	-	No	No	No
Mexico	126 million	nd	>2	nd	0	-	-	-	-	-	-	No	No	No
**Europe**														
Austria	9 million	nd	2	nd	1	2019	0	0	MSc	2.5	7	No	No	Yes
Belgium	11.548 million	nd	25	nd	1	2020	0	0	PGDip	2	5	No	No	No
Cyprus	1 million	nd	2	-	-	-	-	-	-	-	-	No	No	Yes
Denmark	5.9 million	24	43	0	0	-	1^c^	0	-	-	-	No	No	No
France	68 million	>175	227	1	3	2004	0	0	MSc	2	40	No	No	Yes
Germany	83 million	nd	7	-	-	-	-	-	-	-	-	No	No	Yes
Greece	10.640 million	nd	5	-	-	-	-	-	-	-	-	No	No	No
Iceland	387,800	nd	3	-	-	-	-	-	-	-	-	No	No	Yes
Ireland	5.15 million	9	17	-	-	-	-	-	-	-	-	No	No	Yes
Italy	59 million	nd	<10	nd	1	2018	0	0	MSc	2	20	No	No	Yes
Malta	618,000	nd	5	-	-	-	-	-	-	-	-	No	No	Yes
Netherlands	17 million	55	44	4	nd	-	-	-	MAN, MPA	2 (for MAN) to 2.5 (MPA)		Yes	No	No
Norway	5.49 million	40	47	1	1	2001	0	0	MGC	2	5	No	No	No
Portugal	10.3 million	6 trained (3 working as GCs)	4	1	1	2009	0	0	MSc	2	5	No	No	Yes
Romania	20 million	76 trained	76	1	1 (inactive)	2009	0	0	MSc	2	25	No	No	Yes
Spain	47.6 million	70	53	1	1	2008	0	0	MSc	2	6	No	No	Yes
Sweden	10 million	30	40-50	0	1	2021	0	0	MSc, PhD	2	20	No	No	Yes
Switzerland	8.7 million	10	16	0	0	-	-	-	-	-	-	No	No	No
United Kingdom	67.5 million	∼310	322	3	2	1992	0	1	MSc	3	50	Yes	No	Yes
**Asia**														
China	1.41175 billion	Unknown	∼6000	nd	∼3 to 5	2015	∼1	Unknown	PGDip	Unknown	Unknown	No	No	Yes
Hong Kong SAR	7.472 million	nd	<15	nd	3	2010s	0	0	MS, PGDip	1.5 to 2	Unknown	No	No	No
India	1.4 billion	∼76	250	1 MS, 1 GradDip, 2 Cert	4	2007	2	3	MS, MGC	1 to 2	10 to 15	No	No	Yes
Indonesia	270 million	nd	140	nd	2	2006	0	0	MS	1.5 to 2	5 to 20	No	No	Yes
Japan	124.947 million	∼230	356	14	28	2003	0	0	MS, MGC, MPH, PhD	2	80	No	No	Yes
Malaysia	32.7 million	5	10	1	1	2015	0	0	MS	2	md	No	No	No
Philippines	110.343 million	11	18	1	1	2011	0	0	MSc	2	5	No	No	No
Singapore	5.64 million	10	17	0	0	-	-	-	-	-	-	No	No	No
South Korea	51.63 million	12	63	Unknown	3	2015	0	0	MGC	2	md	No	No	Yes
Taiwan	23.4 million	120	198	1	1	2003	0	0	MS	2	13	No	No	Yes
Thailand	71 million	nd	0	nd	2	2022	2	0	Pbacc	4 months	80	No	No	No
**Middle East**														
Egypt	111 million	nd	1	-	-	-	-	-	-	-	-	No	No	No
Israel	9 million	80	150	3	3	1997	0	0	MS	2	6 to 12	Yes	No	No
Jordan	11.508 million	nd	6	-	-	-	-	-	-	-	-	No	No	No
Oman	4.450 million	nd	4	-	-	-	1	0	-	-	-	No	No	No
Qatar	2.985 million	nd	9	nd	1	2018	0	0	MS	2	4	Yes	No	No
Saudi Arabia	35.015 million	20	41	2	2	2014	0	0	MS	2	10	Yes	No	No
Turkiye	85 million	nd	0	nd	1	2020	0	0	MGC	2	2	No	No	No
United Arab Emirates	9.5 million	nd	10	nd	0	-	1 (start 2024)	0	MGC	2	4 to 5	No	Yes	No
**Africa**														
Ghana	30.8 million	nd	0	nd	1	2022	0	0	MS	2	8	Yes	No	No
South Africa	60.756 million	20	30	2	2	1989	0	0	MS	2 (Wits 1y)	2-6	Yes	NA	No
**Oceania**														
Australia	26 million	220	350-440	2	2	1995	1^d^		MS, MGC	2	51	Yes	No	Yes
New Zealand	5.180 million			0	0	-	-	-	-	-	-	No	No	Yes

*MAN*, Master of Advanced Nursing; *MGC*, Master of Genetic Counseling; *md*, missing data; *MPA*, Master of Physician Assistant; *MS/MSc*, Master of Science with specialization in Genetic Counseling; *nd*, no data; *Pbacc*, Postbaccalaureate certificate; *PGDip*, Postgraduate diploma; *PhD*, Doctor of Philosophy; *Wits*, University of Witwatersrand.

a Refer to Table 2 for more information about regulation of genetic counselors.

b Data adapted from Abacan et al^1^ 2019.

c This refers to the planned Joint Nordic Master’s Program in Genetic Counseling by Sweden, Norway, Finland, Iceland, and Denmark.

d This refers to the Graduate Certificate in Genetic Counseling Skills to be offered by the University of Technology Sydney in 2024.

**Table 2:** Regulation of genetic counselors across countries

Country	Internal Self-Regulation by a Professional Organization^a^	Self-Regulation by National Recognition^a^	Statutory Regulation^a^	Relevant Websites
North America				
Canada	CBGC	NA	NA	https://www.cbgc-cccg.ca/?page=1
United States of America	ABGC	NA	Regulation implemented by 35 (out of 50) states	ABGC: https://www.abgc.net/ State licensure of genetic counselors: https://www.nsgc.org/POLICY/State-Licensure-for-Genetic-Counselors
Europe				
Various European countries	EBMG	NA	NA	https://www.ebmg.eu/408.0.html
Netherlands	NA	NA	Registration Committee for Specialties in Nursing (RSV, for Master of Advanced Nursing) Ministry of Health, Wellbeing, and Sports (for Master of Physician Assistant)	RSV: https://www.venvn.nl/registers/verpleegkundig-specialisten-register/het-register/registratiecommissie/ Ministry of Health, Wellbeing, and Sports: https://www.bigregister.nl/herregistratie/criteria-per-beroep/physician-assistant
Sweden	SFMG	NA	NA	https://sfmg.se/arbetsgrupper/genetiska-vagledare/
United Kingdom	NA	GCRAB	HCPC	GCRAB: https://gcrb.org.uk/ HCPC: https://www.ahcs.ac.uk/
Asia				
China	CBGC	NA	NA	https://www.cbgc.org.cn/profile/charters/
India	BGCI	NA	NA	https://www.geneticcounselingboardindia.com/
Indonesia	ISGC^b^	NA	NA	https://inashg.org/about-isgc/
Japan	JSHG and JSGC	NA	NA	https://plaza.umin.ac.jp/∼GC/
South Korea	KSMG	NA	NA	http://www.ksmg.or.kr/html/?pmode=cert3_1&MMC_pid=33
Taiwan	TAGC	NA	NA	http://www.taiwangc.org.tw/
Middle East				
Israel	NA	NA	MoH	https://www.gov.il/en/departments/ministry_of_health/govil-landing-page
Qatar	NA	NA	MoPH	https://www.moph.gov.qa/arabic/Pages/default.aspx
Saudi Arabia	NA	NA	SCFHS	https://www.scfhs.org.sa/en
United Arab Emirates	NA	NA	DHA and DoH	DHA: https://www.dha.gov.ae/en Abu Dhabi DoH: https://www.doh.gov.ae/en/pqr/allied-healthcare-professionals
Africa				
Ghana	NA	NA	AHPC	https://www.moh.gov.gh/allied-health-professions-council/
South Africa	NA	NA	HPCSA	https://www.hpcsa.co.za/
Oceania				
Australia	HGSA	NASRHP	NA	HGSA: https://www.hgsa.org.au/ NASRHP: https://nasrhp.org.au/
New Zealand	HGSA	NA	NA	https://www.hgsa.org.au/

*ABGC*, American Board for Genetic Counseling; *AHPC*, Allied Health Professions Council; *BGCI*, Board of Genetic Counseling India; *CBGC*, Canadian Board of Genetic Counseling; *DHA*, Dubai Health Authority; *DoH*, Abu Dhabi Department of Health; *EBMG*, European Board of Medical Genetics; *GCRAB*, Genetic Counsellor Registration Advisory Board; *HCPC*, Health and Care Professions Council; *HGSA*, Human Genetics Society of Australasia; *HPCSA*, Health Professions Council of South Africa; *ISGC*, Indonesian Society of Genetic Counselors; *JSHG*, Japan Society of Human Genetics; *JSGC*, Japan Society of Genetic Counseling; *KSMG*, Korean Society of Medical Genetics and Genomics; *MoH*, Ministry of Health; *MoPH*, Ministry of Public Health; *NASRHP*, National Alliance of Self-Regulating Health Professions; *RSV*, Registration Committee for Specialties in Nursing; *SCFHS*, Saudi Commission for Health Specialties; *SFMG*, Swedish Society of Medical Genetics and Genomics; *TAGC*, Taiwan Association of Genetic Counseling.

a The classification of regulation used in this table is based on Hoskins et al,^31^ 2021.

b Indonesia will start regulating its genetic counselors through their professional organization in 2024.

**Table 3:** Professional organizations in genetics and genetic counseling worldwide

Country/Region	Organization	Website
**International/Regional organizations**		
Asia	PSGCA	https://psgca.org/
Europe	ESHG	https://www.eshg.org/home
	EBMG	https://www.ebmg.eu/413.0.html
Latin America	SPLAGEN	https://www.splagen.org/
Various countries	Transnational Alliance of Genetic Counseling	http://tagc.med.sc.edu/
Various countries	ASGC	https://www.asgcsociety.org/
**Country organizations**		
**North America**		
Canada	CAGC	https://www.cagc-accg.ca/
	Association des conseilleres et conseillers en genetique de Quebec - Quebec Association of Genetic Counsellors	https://accgq-qagc.ca/
	OAGC-ACGO	https://oagc-acgo.ca/
United States of America	NSGC	https://www.nsgc.org/
	ABGC	https://www.abgc.net/
	ACGC	https://www.gceducation.org/
	AGCPD	https://agcpd.org/
**Central and South America**		
Mexico	AMGH	https://www.amgh.org.mx/
	CMG	https://www.cmgac.org.mx/
**Europe**		
Austria	ÖGH	http://www.oegh.at/
Belgium	BeSHG	https://beshg.be/
Denmark	DSMG	https://dsmg.dk/
France	AFCG	https://af-cg.fr/
Germany	GFH	https://www.gfhev.de/
Italy	Societa Italiana di Genetica Umana	https://sigu.net/
Netherlands	VKGN	https://www.vkgn.org/
Malta	GGCAM	https://maltacvs.org/voluntary/genetic-and-genomic-counselling-association-of-malta/
Norway	NSHG	https://www.nshg.no/
	Norwegian Interest Association for Genetic Conseling	no website
Portugal	APPAcGen	website under review
	SPGH	https://spgh.net/
Romania	Romanian Association for Genetic Counseling	http://www.geneticounselling.ro/
Spain	SEAGen	https://seagen.org/
Sweden	SFGV	https://sfgv.n.nu/
	SFMG	https://sfmg.se/
Switzerland	Swiss Association of Genetic Counselors	no website
United Kingdom	AGNC	https://www.agnc.org.uk/
**Asia**		
China	CBGC	https://www.cbgc.org.cn/
Hong Kong SAR	HKSGC	https://hksgc.org/
India	BGCI	https://www.geneticcounselingboardindia.com/
Indonesia	InaSHG	https://inashg.org/
	ISGC	https://inashg.org/about-isgc/
Japan	JACGC	https://jacgc.jp/
	JSGC	http://www.jsgc.jp/info.html
Malaysia	GCSM	https://gcsocietymalaysia.org.my/
Philippines	PSGC	https://www.facebook.com/PhilippineSocietyofGeneticCounselors
Singapore	GCSS	https://www.linkedin.com/in/genetic-counsellors-society-singapore-2aa2a429a/?originalSubdomain=sg
South Korea	KSMG	http://www.ksmg.or.kr/html/?pmode=cert3_2&MMC_pid=33
Taiwan	Taiwan Association of Genetic Counseling	http://www.taiwangc.org.tw/
Thailand	MGGA	https://tmgga.org/index.php
**Middle East**		
Israel	Israeli Association of Genetic Counselors	no website
Oman	Oman Society of Medical Genetics	no website
Saudi Arabia	Saudi Network of Genetic Counselors	no website
	SSMG	https://ssmg.org.sa/en
Turkiye	TGD	https://www.tibbigenetik.org.tr/
**Africa**		
South Africa	GC-SA	https://sashg.org/about/focus-groups/genetic-counsellors-south-africa/
**Oceania**		
Australia and New Zealand	HGSA	https://www.hgsa.org.au/
	ASGC	https://www.hgsa.org.au/ASGC/Default.aspx

*ABGC*, American Board of Genetic Counseling; *ACGC*, Accreditation Council for Genetic Counseling; *AFCG*, French Association of Genetic Counselors; *AGCPD*, Association of Genetic Counseling Program Directors; *AGNC*, Association of Genetic Nurses and Counsellors; *AMGH*, Asociacion Mexicana de Genetica Humana; *APPAcGen*, Associacao Portuguesa dos Profissionais do Aconselhamento Genetico; *ASGC*, Australasian Society of Genetic Counselors; *ASGC*, Arab Society of Genetic Counselors; *BeSHG*, Belgian Society for Human Genetics; *BGCI*, Board of Genetic Counseling India; *CAGC*, Canadian Association of Genetic Counsellors; *CBGC*, Chinese Board of Genetic Counseling; *CMG*, Consejo Mexicano de Genetica; *DSMG*, Danish Society for Medical Genetics; *EBMG*, European Board of Medical Genetics; *ESHG*, European Society of Human Genetics; *GC-SA*, Genetic Counsellors South Africa; *GCSM*, Genetic Counselling Society Malaysia; *GCSS*, Genetic Counsellors Society Singapore; *GGCAM*, Genetic and Genomic Counselling Association of Malta; *GFH*, German Society of Human Genetics; *HGSA*, Human Genetics Society of Australasia; *HKSGC*, Hong Kong Society of Genetic Counseling; *InaSHG*, Indonesian Society of Human Genetics; *ISGC*, Indonesian Society of Genetic Counselors; *JACGC*, Japanese Association of Certified Genetic Counselors; *JSGC*, Japanese Society for Genetic Counseling; *KSMG*, Korean Society of Medical Genetics and Genomics; *MGGA*, Medical Genetics and Genomics Association; *NASRHP*, National Alliance of Self-Regulating Health Professions; *NSGC*, National Society of Genetic Counselors; *NSHG*, Norwegian Society of Human Genetics; *OAGC-ACGO*, Ontario Association of Genetic Counsellors-Association des conseiller-ere-ær-s genetique de l'Ontario; *ÖGH*, Austrian Society for Human Genetics; *PSGC*, Philippine Society of Genetic Counselors; *PSGCA*, Professional Society of Genetic Counselors in Asia; *SEAGen*, Spanish Society of Genetic Counseling; *SFGV*, Swedish Association for Genetic Counselors; *SFMG*, Swedish Society of Medical Genetics and Genomics; *Sigu*, Italian Society of Human Genetics; *SPGH*, Sociedade Portuguesa de Genetica Humana; *SPLAGEN*, Latin American Professional Society of Genetic Counseling; *SSMG*, Saudi Society of Medical Genetics; *TGD*, Society for Medical Genetics; *VKGN*, Association of Clinical Genetics Netherlands.

### Growth of the profession

Since the article by Abacan et al,^1^ the number of genetic counselors globally has increased significantly to an estimate of greater than 10,250 genetic counselors in nearly 50 countries at the end of 2022. This is largely related to increases in the number of US genetic counselors (from 4000 to >6500). Many countries showed an approximate doubling of their GC numbers during the past 5 years. Twenty new countries have been documented as having genetic counselors practicing clinically since the last report. Notably, a high percentage of these describe that the first genetic counselors were trained internationally, most often at accredited programs in Australia, Canada, the United Kingdom, and United States.

Much of the increase in genetic counselor numbers or in listed opportunities for growth in the next 5 years revolve around country-wide sequencing projects or biobanks (Hong Kong SAR, Qatar, Saudi Arabia, and Unied Arab Emirates [UAE]) or large national carrier screening or newborn screening programs (Australia, Oman, Philippines, Qatar, Saudi Arabia, and UAE).

“The number of genetic counselors has increased at least 2 times since the Hong Kong Genome Project launched in 2021. There is the rapid need for genetic counselors providing pre-test and post-test counseling for the participants enrolled in the Project.”

“The biggest opportunity is still the increasing demand from several fronts- primarily newborn screening which plans to have at least one genetic counselor per province (the Philippines has 81 provinces).”

There has also been an increase in roles such as laboratory genetic counselor positions and clinical specialization. Mainstreaming of genetics and genomics has been defined elsewhere as a model involving “genetic nurses and physicians identifying at-risk individuals and initiating genetics discussions by integrating genetics into practice.”^36^ Many participants commented specifically on how genetic counselors assist with this process through expanding their roles to work directly with nongenetics medical specialists, although in some places this was primarily described as working directly with oncologists.

“Clinician scientists in various disciplines are hiring GCs (rare disorders, cardiogenetics, neurogenetics, etc.).”

“Within NHS positions, a greater proportion of Genetic Counselors have an established specialty interest area (eg, cancer, cardiac, renal, endocrine, prenatal, PGT, immunogenetics, ophthalmology, neurology, metabolic, pediatric). This has become more necessary as the complexity of the cases seen has increased. Specialty knowledge enhances the standard of care and the autonomy of practice.”

A smaller number of countries specified that their genetic counselors work primarily or exclusively with medical geneticists or in laboratories:

“GCs still practice primarily independently in Divisions that include clinical geneticists. GCs occasionally see pediatric/adult patients jointly with clinical geneticists. No mainstreaming (yet).”

It is also important to note that as genetic counselor training remains, for the moment, broad in some locations and as roles expand beyond clinical settings, there is a fuzzy line of how individuals calling themselves genetic counselors may be defined, as illustrated by this respondent:

“I have noticed more individuals using the title of “genetic counselor” while working in non-traditional roles such as signing laboratory results or serving as a laboratory manager. However, I am uncertain whether they fully understand the traditional role of a genetic counselor.”

We asked respondents how GC services had changed since the COVID-19 pandemic. Many commented on the useful integration of telehealth into regular services, addressing geographic, time, and mobility barriers for patients. For some genetic counselors this has also increased remote work opportunities.

“With the pandemic, telephone consultations were introduced and are still utilized alongside in person consultations. … COVID-19 required us to depend overwhelmingly on telephone consultations. Post-COVID-19, telephone consultations/counseling has become a preferred option for some patients. Telehealth has helped us increase access to genetic counseling services in cases of disease-related ambulation difficulties, lack of transportation, work/school commitments, etc.”

Finally, respondents have commented that the increase in the number of genetic counselors and the presence of more training programs have, in general, facilitated awareness on the value and access to GC services and allowed genetic counselor numbers to increase.

“The increase in number of GCs led to a significant expansion of the field of genetic counseling and the roles GCs hold. This is helping increase awareness among non-genetics healthcare providers, increase referrals, and increase the number of patients/families served. Ultimately, access to genetics/GC services increased and our training capabilities increased.”

“Genetic counselor graduates have outpaced geneticist training and therefore are more accessible across health systems. As large systems have been created, genetic counselors have the opportunity to impact the health care providers in appropriate identification, risk assessment, initial testing and triage toward meeting with geneticist or other appropriate specialist. We can be the primary genetic resource.”

### GC training

We identified a total of 136 GC training programs across 30 countries, nearly half of which are in North America (Table 1). Nearly all GC training programs around the globe are master’s-level programs. Postbaccalaureate certificate or a postgraduate diploma/certificate in GC is offered in a few countries, such as in Belgium, Chile, China, Hong Kong SAR, and Thailand. There has been a dramatic increase in the number of GC training programs globally in the past 5 years—in the United States, for example, the training programs went from 39 to 56; in Japan, they went from 14 to 28.

We were able to document earlier programs in China (2015), Hong Kong SAR (2010s), Indonesia (2006), and Saudi Arabia (2014) that had not been previously reported by Abacan et al.^1^ New programs started in Austria (2019), Belgium (2020), Ghana (2022), and Italy (2016 as a certificate program for nurses and genetic counselors and moving to a master’s program in 2018), Qatar (2018), Sweden (2021), and Turkiye (2020). They are actively upcoming in UAE (2024) and Oman (2025). Programs have closed or were put on hold in Hong Kong SAR, Romania, the United Kingdom (Glasgow), and the United States (Brandeis). In Spain, 1 program closed and reopened at a new university. Some 1-year programs have closed, and new 2-year master’s programs have started in India.

Countries that are new to establish GC and GC programs describe the difficulty to provide clinical training because there are a dearth of clinical supervisors with GC training. In these cases, the clinical training is frequently supervised by medical geneticists.

“Most students currently learn from medical geneticist[s] but we try hard to also provide experience with trained GCs.”

As countries become more established with the profession of GC, they often comment that the clinical supervision transitions toward utilizing more master’s-trained genetic counselors in the training process.

“We have 7 genetic counselors who have graduated from an international program (5 from the UK and 2 from the US), and now more genetic counselors who have completed a local program are working in different institutes and in different clinical areas, such as cancer genetics, allowing more clinical training sites for graduates and greater experience.”

Regarding the required research for the degree, several commented that the required research to complete the GC degree is often either laboratory or clinically focused (eg, description of clinical features) research, particularly in countries where GC is still an emerging profession.

“[a]cademic institutes and Universities still are not ready for research in topics relevant to genetic counseling and prefer experimental based research.”

This is in contrast to learning that there are several doctoral level programs specifically for or relevant to GC research in Australia, United Kingdom, United States, South Africa, Japan, and Sweden. As illustrated in the quote below, this is beginning to establish a more academic and research-focused culture in the profession.

“There is a growing number of genetic counselors with a PhD in Australasia, creating a strong research culture in the profession.”

As described above, in many countries, new GCs are trained in locations with long-standing GC education programs (eg, Australia, Canada, United Kingdom, and United States) to come back and seed the profession in a country where GC has not yet developed. Training in Austria, France, and Italy also play a role in spreading the profession across multiple countries in Europe that share the language of training. In other places, people simply start taking on the roles associated with a genetic counselor and then consider getting official training (eg, Jordan, other places).

Finally, training models vary in countries where GC is developing, especially if there are regulations about which types of providers can offer GC. For example, in some countries, GC training is provided for healthcare providers who already have a legal basis for practice (nurses, physician assistants, or even trained physicians). In these countries, such as Belgium, Chile, China, and Thailand, healthcare providers are trained and given a diploma or a postbaccalaureate certificate in GC, but even these programs are being considered to be elevated into full-fledged master’s programs.

“We give priority to those who already have a health care profession according to Belgian law (nurse, midwife or psychologist) as genetic counselor is not a recognized health care profession in our country. To those who do not have a health care profession we stress at the start of the program that the certificate does not guarantee them a future position.”

We also note that, with the advent of short courses in genetic and genomic medicine that are offered to healthcare professionals, it has been increasingly difficult to define the professional title of genetic counselor on a consistent global basis.

“Previously, it was a widely held belief that there were no genetic counselors in Thailand. However, the current number varies depending on the criteria used for counting. If the definition is limited to those holding a master’s degree in genetic counseling, then there is only one. On the other hand, if we include healthcare professionals who have completed the four-month course in genetic counseling, the number increases to approximately 60-80 individuals.”

### Professional regulation of genetic counselors

Across the globe, genetic counselors are regulated in different ways; some governmental regulations also include title protection (for example in some US state licensure bills). Many countries have a credential that is available through a professional body (Table 1, Table 2 and Table 1, Table 2). Only a few countries have established national (and in some cases statutory or law by a legislative body) regulation of genetic counselors (Australia, Ghana, Israel, The Netherlands, Qatar, Saudi Arabia, South Africa, United Kingdom; Table 1, Table 2 and Table 1, Table 2). Two have state or provincial regulation (UAE and United States), and many are actively working on national regulation and a legal basis for the profession.

Countries that do not yet have national regulation of the profession frequently cited this as one of the biggest challenges for the profession within their country.

“The biggest challenge remains to obtain official recognition at a federal level (recognition of the profession as a health profession) and the recognition/acceptance from the … genetics society.”

In many countries, only MDs or other registered providers are able to provide GC (eg, Mexico, Guatemala, Philippines, S Korea, The Netherlands, and Thailand). For some (The Netherlands, Philippines, and Turkiye), this has specifically influenced their training approaches.

“… genetic counselors are not legally recognized as a distinct profession. The current legal framework acknowledges only healthcare professionals (HCPs) like nurses, doctors, and medical technologists, who have received specialized training in genomic medicine, as qualified to practice in areas including genetic counseling.”

“In some countries, the increase in numbers of GCs that allow the formation of a professional society of GCs was mentioned as a first step in being able to advocate for regulation or recognition of the profession.”

“We still do not have a mechanism in place to regulate the practice of genetic counselors. However, the establishment of the Philippine Society of Genetic Counselors is a first step to establish [a] mechanism for self-regulation.”

Lastly, some respondents also mentioned a desire for a cross-country regulation across Arabic-speaking countries, for example.

### Billing and reimbursement

This question was interpreted differently by respondents and provided different types of data.

Some respondents chose to comment on whether genetic counselor services were reimbursed. Some countries stated that services were covered as part of a national healthcare system.

“...genetic counseling services are mainly offered at NHS and keep the same public reimbursement rules, free at the point of delivery if a referral exists.”

Others (eg, the United States) mentioned that they had obtained the ability to bill some healthcare payers for GC provided by genetic counselors.

“CPT code 96040 has been established since 2007 and is reimbursed by some private insurance companies but not at a level that sustains genetic counselor clinical practice. Medicare/Medicaid funded patients are not allowed to bill for genetic counseling.”

Finally, there were countries where the only ability to bill was by physicians or other approved healthcare providers.

“Reimbursement is still only available for genetic counseling conducted by a physician after genetic tests.”

Salary of genetic counselors was the second related area that respondents commented on. Some mentioned that there were not any salary levels associated with genetic counselor job descriptions and that therefore the salary assigned was generally based on the prior qualifications that existed before GC training.

“...reimbursement is strongly dependent on the circumstances and prior qualification (BSc, MSc, PhD as prior qualifications).”

Several countries reflected that genetic counselor salaries were increasing, at least for some.

“The starting amount has increased marginally, but the experienced GCs have double the salary of what they were getting 5 years back.”

“Recent pay equity reviews … have recognized the training and specialization of the GC role resulting in salary increases nationally.”

### Brief summaries by region

#### North America (∼7100 genetic counselors)

The United States and Canada have very well-established GC professions. Over the past 5 years, the GC profession has grown significantly (∼65%) in North America, from approximately 4400 in 2017 to over 7100 genetic counselors and 61 GC training programs between the 2 countries at the time this article was written. GC programs are primarily master’s level training, with some emerging doctoral level research-focused programs in the United States. Genetic counselors in both countries have professional credentialing bodies (American Board of GC, [ABGC], and Canadian Board of Genetic Counselling, [CBGC]), and the Accreditation Council of GC (ACGC) reviews and accredits programs in both countries. There is some ability for genetic counselors trained outside of North America to obtain ABGC or CBGC certification, but these options are currently limited to genetic counselors who are already registered with an approved organization, and individuals still have to pass a certification examination.^37^ ABGC had 6517 certified genetic counselors in March 2023 and currently lists more than 6800 certified genetic counselors. The CBGC listed 380 certified genetic counselors in February 2024.

There has been growth in the number of genetic counselors working at laboratories and in a wide range of clinical specialties outside of medical genetics units. Many GC jobs are now remote, particularly given the growth in tele-GC. Both the United States and Canada report that one of the greatest current challenges is professional regulation of genetic counselors. Some Canadian provinces have local or hospital-based regulation, and 35 of 50 US states have established licensure,^38^ but neither country has national regulation or recognition in place as of 2023. Notably, the GenCOUNSEL research project in Canada^39^ and the US-based NSGC have been working toward national recognition and regulation for several years.

#### Central and South America (estimated ∼500 genetic counselors)

Cuba is the one country that has a profession of GC. The Abacan et al^1^ article reported approximately 900 genetic counselors in 2017; an update determined that there are currently 474 master’s-level genetic counselors and 104 medical geneticists. All are operating under the organization of the National Center for Medical Genetics and work at 3 levels of public healthcare directed by the Ministry of Public Health. The significant decrease in numbers of genetic counselors is attributed to people leaving the field because of retirements, emigration, and COVID deaths.

Genetic counselors are still rare in other countries across Central and South America; many countries still have legal requirements that medical care is provided by physicians. A master’s-level GC training program started in Brazil in 2015, and its website lists ∼20 graduates,^40^ but we were not able to learn more about whether the graduates are working as genetic counselors. There are a handful of genetic counselors with international master’s level training in GC who are working in Chile, Guatemala, and Mexico. Some countries are trying to educate healthcare professionals to address the demand for GC; for example, in Chile in 2022, there was a Diploma in GC that educated 41 healthcare professionals, and they are actively exploring the feasibility of launching a master’s Degree in Cancer GC. There was also a workshop focused on GC at the 2022 Human Genome Organization meeting in Brazil, raising awareness regarding the profession and teaching GC skills to healthcare providers.

An important step toward expanding GC services in Latin America was the 2022 development of the SPLAGen. Founded by US-based genetic counselors, SPLAGen has goals of connecting genetics professionals, networking, education, advocacy, and research around GC services in Central and South America. They have compiled lists of providers and collected Spanish language genetics educational material, among other projects.^41^ Some countries have also published a needs assessment about GC practice (eg, Mexico^42^).

#### Europe (∼960 genetic counselors)

We estimate that there are ∼960 genetic counselors in Europe. Although the number of genetic counselors in Europe has not changed dramatically in the past 5 years, there are now genetic counselors in at least 19 European countries: Austria, Belgium, Cyprus, Denmark, France, Germany, Greece, Iceland, Ireland, Italy, Malta, Netherlands, Norway, Portugal, Romania, Spain, Sweden, Switzerland, and the United Kingdom. There are a handful of US-trained genetic counselors living in other European countries but generally not providing clinical services within Europe; some provide services via US-based genetics companies, and others have taken on other roles, such as research, clinical trials coordination, or related work areas. There is 1 recent publication that has examined the perceived role of the genetic counselors in European genetics clinics.^43^

At the end of 2023, there were 12 active training programs across Europe (Austria, Belgium, France [3 programs], Italy, Norway, Portugal, Spain, Sweden, and the United Kingdom [2 programs]). All are master’s-level programs except for 1 graduate diploma program in Belgium,^44^ which prioritizes training those with healthcare professions (eg, nurse, midwife, and psychologist). Notably, during the past several years, the MSGC training programs in The Netherlands have closed, and the training has been subsumed under Master’s of Physician Assistant and Advanced Nurse Practice degrees because these providers are able to legally practice independently. The program in Romania has been inactive for several years, and 1 program in France is expected to close.

One major change with genetic counselor training in Europe is that, since 2016 in England, United Kingdom, there is a commissioned 3 year scientiﬁc training program in Genomic Counseling with an annual intake of 25 students (up from 15 places when the course began). The scientiﬁc training program combines a 3 year MSc in Genetic and Genomic Counseling with work-based training competencies in line with the Genetic Counsellor Registration Advisory Board (GCRAB) GC Competencies and Academy for Health Care Scientists “Good Scientific Practice.” Successful completion of the master’s degree and work-based competencies with a final Independent Assessment of Clinical Competencies interview awards Statutory Regulation with the Health and Care Professions Council. The voluntary registration board previously, the UK Genetic Counsellor Registration Board has joined the Academy for Healthcare Science as the profession moves toward Health and Care Professions Council statutory registration through a process of equivalence. This process of equivalence also includes the devolved nations of Scotland, N. Ireland, and Wales. A second MSc GC program in the United Kingdom is a part-time 3-year MSc in Genetic and Genomic Counseling at Cardiff University in Wales. Trainee genetic counselors from an MSc background and nursing/midwifery backgrounds continue to register with the GCRAB as the profession works to increase workforce through multiple entry routes maintaining the professional skills mix. Future apprenticeship schemes are also being explored improving diversity and inclusion into entry routes for the profession. A growing Genomic Associate workforce is also supporting entry into training. Growth in private practice and “mainstream” genetic counselors is also observed in the United Kingdom.7 The UK Genomics Strategy aims to embed genomics across National Health Service care with a centrally funded genomic test directory. Commissioning genetic services and workforce planning continue to drive the need for increasing training positions and potential growth of master’s programs.

Credentialing in the United Kingdom (and the Republic of Ireland) occurs separately through GCRAB. The European Board of Medical Genetics (EBMG) provides program accreditation and genetic counselor registration across the rest of Europe, including an abbreviated registration pathway for genetic counselors working in Europe who trained elsewhere and have up to date credentials from other approved credentialing bodies. UK genetic counselors who have GCRAB registration are also eligible for EBMG credentials. As of the writing of this article, 129 genetic counselors have been registered by EBMG.^45^ There are currently 322 genetic counselors registered in the United Kingdom. Notably, as part of the CAN.HEAL consortium, there is an ongoing project that has recently reviewed European Union Member State legislation and regulation as they related to GC practice.^46^

#### Middle East (∼220 genetic counselors)

There are now approximately 220 genetic counselors in the Middle East. Israel and Saudi Arabia still account for the majority of genetic counselors in this region, both of which noted an approximate doubling of the numbers in the past 5 years (from 80 to 150 and 20 to 41, respectively). However, there are many new countries (Jordan, Oman, Qatar, and UAE) reporting the profession of genetic counselors and several new master’s-level training programs developed and in process (Qatar 2018, Turkiye 2020, UAE starting in 2024; Oman planned for 2025). We also note a US-trained genetic counselor has started practicing clinically in Egypt in 2023, providing online services through a genetics company for patients in Egypt, Jordan, and Lebanon (among other areas). Jordan and Oman serve as useful examples of locations where the genetic counselor profession is emerging by following different paths. In Jordan, a US-trained genetic counselor has been actively practicing since 2010, establishing the first GC clinic in Jordan that serves the North and Middle regions in the country and expanding in 2018 to Amman. Her practice encompasses a wide range of GC services, including premarital, pediatric, cancer, adult, metabolic disorders, neurology, and cardiology GC, as well as working as a lab genetic counselor. Of the 6 genetic counselors reported in Jordan, 2 will have graduated from accredited GC programs (1 as described above and 1 in 2024). These 2 genetic counselors are entitled to perform comprehensive pre- and postgenetic testing counseling sessions in a range of clinical areas. Most GC happens with medical specialists because of the dearth of medical geneticists in Jordan. The remaining Jordanian genetic counselors with non-GC degrees typically perform more basic tasks, such as taking family histories, preparing samples, and reporting results to physicians. In contrast, the genetic counselors in Oman have primarily received (or will receive) master’s-level genetic counselor training internationally. They report their biggest job opportunity is related to the expansion of the genetic services of rare diseases diagnosis, availability of genetic testing, and the national premarital screening program for common blood disorders.

Israel has the most well-developed framework on GC practice in the region, with national professional regulation^47^ and 3 master’s-level training programs. They report that genetic testing is moving away from founder variant testing and that cancer genetics is moving into a mainstreaming approach. They also report a high demand for laboratory genetic counselors working in variant analysis. Saudi Arabia has national professional regulation by the Saudi Commission for Health Specialties (SCFHS).^48^ Since 2022, Qatar also has a national professional regulation,^49^ and all genetic counselors are required to hold a master’s degree in GC.

Finally, the Arab Society of Genetic Counselors^50^ was founded by US-based Arab genetic counselors in 2019 to connect genetic professionals serving Arab patients around the world.

#### Asia (estimated ∼1070 genetic counselors)

GC has expanded significantly in Asia during the past 5 years with ∼1070 genetic counselors in Asia outside of China. Large jumps were noted in the numbers of genetic counselors in Japan and Taiwan (50% increase), and more than doubling in India and South Korea. In addition, genetic counselors are reported in Hong Kong SAR, Indonesia, Malaysia, Philippines, Singapore, South Korea, Republic of China, and Thailand. We have received reports of between 4000 to 6000 genetic counselors in China, but as is noted below, their training is significantly different from what is reported in other countries.

GC training programs in Asia exist in China, Hong Kong SAR, India, Indonesia, Japan, Malaysia, Philippines, South Korea, Taiwan, and Thailand.^32^ Notably, the number of GC training programs in Japan has doubled from 14 in 2017 to 28 in 2022; Japan also offers a doctoral program in GC. Training programs are primarily master’s level across Asia, with 2 countries offering postgraduate diplomas (China and Hong Kong SAR) and a postbaccalaureate certificate in Thailand. Because they are somewhat unique, they warrant some discussion. Thailand currently does not have a master’s-level training program but a 4-month short course in medical genetics and GC that awards a postbaccalaureate certificate. Offered by 2 institutions in Thailand, these short courses have trained the first cohort of 20 healthcare providers (ie, physicians, nurses, and medical technologists) in 2022 and currently, discussions are underway to upgrade these courses into full-fledged master’s programs. In China, genetic counselors are certified after completing a short training (equivalent to 1-2 week course).^51^^,^^52^ As such, the number of genetic counselors reported in China (up to 6000) cannot necessarily be considered equivalent to the numbers shown in other countries.

Five countries in Asia (China, India, Japan, South Korea, and Taiwan) have established certification mechanisms through a professional organization, whereas 2 countries (Indonesia and Malaysia) have ongoing discussions with their respective ministries of health for credentialing of genetic counselors. The Professional Society of Genetic Counselors in Asia (PSGCA), founded in 2015, continues to be a significant avenue for Asian genetic counselors to collaborate and exchange resources.

#### Africa (∼30 genetic counselors)

Genetic counselors on the African continent are still primarily centered in South Africa. There are currently ∼30 genetic counselors compared with the ∼20 reported previously. More counselors have been trained but many have immigrated and are employed in other countries. This remains the biggest challenge in the country because job opportunities are few.^53^ The lack of jobs and the development of technologies have resulted in genetic counselors moving into private laboratories or setting up private practice. There are now more counselors working in the private sector (∼2/3) compared with those working in state services.

There are 2 master’s GC programs in South Africa (The University of Witwatersrand and the National Health Laboratory Service [Wits] and the University of Cape Town).^53^ The 2 programs graduate between 4 to 10 students per year; the difference in numbers is because Wits has an intake every 2 years. University of Cape Town also offers a PhD in GC, entry is a master’s in GC. In the past year, Wits converted their 2-year master’s degree to a 1-year degree because of pressure from the university to convert all master’s degrees to 1-year programs. Their first intake will be in 2024. The graduates will however still undergo 3 years of training (1 year academic and 2 years of internship training) in accordance with South African legislation. Ghana has started a new GC program in 2022, developed by scientists and a psychologist in conjunction with the 2 programs in South Africa. The program was developed because of a great need for providing GC to individuals and families as an adjunct to genomics research in Africa. The program is a 2-year master’s degree and enrolled 8 to 12 students in 2022 and 2023. Beyond these GC training programs, the African Genomic Medicine Training Initiative was established to provide training to healthcare professionals, other than genetic counselors and medical geneticists such as nurses, doctors, and pharmacists in Africa.^54^ It is the first community-based blended learning course for nurses across 11 African countries, with the first intake in 2019.

The Health Professions Council of South Africa is the regulatory body in South Africa, and all graduates are required to register as an Intern in GC and complete 24 months of training in an accredited facility before being eligible to submit a portfolio of evidence for assessment.^55^ Only upon passing the assessment can the intern register as a genetic counselor in independent practice and start providing a service. Genetic counselors are able to charge for their service because there are established billing codes, and they are reimbursed by medical aids/insurance. The Genetic Counselors of South Africa professional group was started in 2009 as a focus group of the South African Society of Human Genetics.^56^ The Genetic Counselors of South Africa works closely with the Health Professions Council of South Africa to provide guidelines and other input related to the practices of Genetic Counselors. Upon completion of their training, Ghana graduates will register with the Psychological Council of Ghana or another regulatory body to enable them to practice in the country.

#### Oceania (>400 genetic counselors)

Genetic counselor numbers have approximately doubled in Australia and New Zealand over the past 5 years, now estimated at nearly 450 in clinical practice. Notably, a related article in this special issue estimates Australasia to have 630 graduates from genetic counselor programs.^57^ In the past 5 years, there is an observed increase in the number of genetic counselors working in mainstreaming, such as in transplant medicine, nephrology, and hematology. Similarly, a growing number of genetic counselors have assumed roles in education and research with some leading grant funded research teams. The increasing number of PhD-prepared genetic counselors in Australia and New Zealand has been reported to create a strong research culture within the profession.

There continue to be 2 master’s-level GC training programs, both in Australia. In addition, a graduate certificate focusing on GC skills is planned to be offered in 2024 by the University of Technology Sydney. This new course is envisioned to equip those already registered as allied healthcare professionals to incorporate competencies in genetics and genomics into their area of practice.

GC training accreditation and genetic counselor credentialing is available through the Human Genetics Society of Australasia (HGSA). The HGSA register currently lists 357 certified genetic counselors.^58^ Aside from self-regulation through the HGSA, Australian genetic counselors are also nationally recognized by being full members of the National Alliance of Self-Regulating Health Professions; New Zealand is working toward a similar pathway. The National Alliance of Self-Regulating Health Professions aims to maintain the quality of health professionals by ensuring that members are satisfying national regulatory requirements.^31^

## Discussion

This article provides a summary of the global GC profession with responses to the survey from over 45 countries. In this article we have looked at areas of development and growth of the GC professional the past 5 years. We document over 10,250 genetic counselors and over 130 genetic counselor training programs, primarily at the master’s degree level. Notably, there are a number of important limitations to these data that are worth discussion. First, the survey was conducted only in English, which may have led to some misunderstandings about content (for example, in regard to the question about billing and reimbursement) and to a loss of important nuance. Second, our sample was essentially a convenience sample; therefore, although we did our best to ensure that we were obtaining all the relevant data, it is possible that we missed some countries or GC training programs that exist globally despite our best efforts. Third, it is possible that our data, which are primarily self-reported, have inaccuracies. We did our best to validate the data through publications or websites and to offer all informants the opportunity to review the article for accuracy before submission in order to address this issue. Finally, and perhaps most importantly, there are still differences in who is defined as genetic counselors globally, and few countries collect annual data in the form of a “professional status survey,” making it difficult to gather consistent data. For example, we noted that some country informants referenced numbers of trained genetic counselors versus practicing genetic counselors or even separated this into those practicing clinically versus in total and this varied by country. Other country informants provided information on medical providers who function as genetic counselors, rather than non-MD genetic counselors. These challenges in defining who a genetic counselor is and what qualifications are required to call oneself a genetic counselor (“title protection”) are significant. More work is needed in this area to provide the most accurate assessment of the global genetic counselor profession in the future.

In our data, we noted several important trends for the profession of genetic counselors. First, the roles of genetic counselors continue to expand clinically in a range of ways. The opportunities for GCs to help facilitate widespread and equitable access to genomic testing was mentioned by almost all survey respondents. Respondents also recognized the way GCs could be effectively embedded in multidisciplinary teams as genomic medicine becomes increasingly and rapidly relevant throughout healthcare. Government recognition, regulation (including title protection) and a consistent funding stream were considered essential to help a country “grow” the genetic counselor profession and to facilitate the introduction of master’s-level GC training programs nationally where these do not yet exist. It was also clear that more data are needed on how genetic counselor services are reimbursed and how this affects the ultimate number of genetic counselor jobs that exist and their status.

One additional point that we noted is the trend toward more countries offering doctoral programs in GC. The increase in doctoral programs mirrors the focus on GC specific research over the past decade or more and warrants more study to better describe what is actually occurring. As was discussed by Dr Austin at the World Congress of GC in October 2023,^59^ this begins to move GC toward a position as an academic discipline as the profession broadens the existing body of literature, develops theories that underlie the work, and clarifies the tenets that may be consistent across the globe. We look forward to watching this evolve further in the upcoming decade.

Finally, there is currently no international organization for the professional development of genetic counselors, and we encourage the consideration of one in future years. Potential roles of such an organization would be to maintain international connections among genetic counselors and their professional organizations, to allow easier international referrals for patient and family follow-ups (for example, cascade testing), and to facilitate and standardize the collection of data similar to this article. There are many different mechanisms by which this might occur. One option would be to develop an independent umbrella organization of GC professional societies, similar to the structure of the International Federation of Human Genetics Societies (www.ifhgs.org). International Federation of Human Genetics Societies also has the option for affiliate memberships of “... national or international organization[s] representing a specialty dedicated to human genetics,” and we note that 2 GC organizations (NSGC and CAGC) are already members. We hope that the recent international discussions described by Campbell and Pirzadeh-Miller (2024)^60^ will facilitate such consideration in upcoming years.

In conclusion, in this article, we have described significant evolution of the profession of genetic counselors across the globe in the past 5 years—increases in numbers of genetic counselors and their roles, as well as in the number of training programs that exist. Although the establishment of the GC profession appears nuanced to the country and its wider socio-political context and history, the survey responses reveal many areas of commonalities including a firm commitment to training, shared values and hopes for the future. To envision clear goals for the profession in the next 5 years, we hope that genetic counselors internationally will come together to assist each other, particularly in terms of establishing further master’s-level training programs, achieving statutory regulation, and developing the financial commitment to support the workforce.

## Data Availability

The quantitative survey data are entirely included in the tables that are published with the article or in supplemental materials. Readers with questions about any of the open-ended comment data are encouraged to contact the corresponding author.

## ORCIDs

Kelly E. Ormond: http://orcid.org/0000-0002-1033-0818

Peter James Abad: http://orcid.org/0000-0002-8899-9044

Rhona MacLeod: http://orcid.org/0000-0001-9239-4526

Masakazu Nishigaki: http://orcid.org/0000-0001-7589-6487

Tina-Marié Wessels: http://orcid.org/0000-0002-2676-0564

## Conflict of Interest

All authors are members of the Board of Directors of TAGC. The authors do not have other conflicts of interest to declare.

## Footnotes

a In this article, we acknowledge that both "genetic counseling" and "genetic counselling" are used across the globe. For simplicity, we will use "genetic counseling" throughout the article unless we are referring to an organization name or degree name that utilizes the alternative spelling.
