# Supplementary material for: The global status of genetic counselors in 2023: What has changed in the past 5 years?
Source: Genet Med Open. 2024 Aug 8;2(Suppl 2):101887. doi: 10.1016/j.gimo.2024.101887 (PMC11658554; doi:10.1016/j.gimo.2024.101887)
Supplement: Supplementary Material [file mmc1.pdf]

Default Question Block

Dear TAGC colleagues,

As you may remember, in 2019 we published a paper on the Global State of Genetic Counseling (Abacan, et al.), based primarily on data collected in 2016 and 2017. As it's been 5 years, the TAGC Board of Directors approved a team to collect new data in order to update this publication.

The research team below has developed a survey that should take approximately 30 minutes to complete and we ask that you work with the appropriate colleagues in your own countries to provide answers regarding the issues below. It is possible that you may be able to complete the entire survey on your own, but it's also possible that you may need to consult with other GC leaders in your country (e.g. presidents of professional organizations, accrediting or credentialling bodies, etc).

We will share a PDF version of this survey so that you can review the questions and obtain answers in advance, and ask that you enter your answers into this survey no later than OCTOBER 15, 2023.

Our plan is to submit this paper for the Genetics in Medicine Open Special Issue on International Genetic Counseling, and we will formally acknowledge anyone who completes the survey

and lists their name (or the name of others who assist in providing data).

As this survey does not collect data about individual persons, it is exempt from Human Subjects Review and we have not submitted it to an ethics committee.

Research team: Kelly Ormond, TAGC president; Peter Abad (Philippines), Rhona MacLeod (UK), Kazu Nishigaki (Japan), Tina Wessels (South Africa)
For questions: kelly.ormond@hest.ethz.ch

What country are you responding from?

How many people are estimated to live in your country in 2022? (please check at this website for consistency: https://www.nationsonline.org/oneworld/population-by-country.htm)

How many genetic counselors do you estimate are working in your country in 2022?

Provide a source for this estimate (e..g. A publication (PDF/doi) or URL from a professional group, or a personal communication, in which case give the name and date)

Please list the professional organizations in your country and add a website/link when possible so that we can add it to the TAGC website (e.g. NSGC, HGSA, CAGC).

Do you have genetic counseling training programs in your country?

- Yes
- No, but they are in active development
- No

What year did genetic counseling training start in your country?

How many training programs are currently operating in your country

We hope to update the TAGC website with the most correct links to training programs in your country. Please check the website listed below (under "Education Programs") and if there are any websites that need to be added or deleted, please list the URLs below.
https://sc.edu/study/colleges_schools/medicine/centers_anc

Are there additional training programs in development? (please list numbers and describe status and any contacts that would be useful for us to know about)

Have any training programs closed in the past 5 years? (please list numbers, reasons)

What degrees are given out for genetic counselors trained in your country? (check all that apply)

- ☐ Master of Science (MS, MSc)
- ☐ Masters in Genetic Counseling (MGC)
- ☐ MPH
- ☐ MPA
- ☐ Doctoral Degree specifically in genetic counseling
- ☐ Post-baccalaurete Certificate or Graduate Diploma
- ☐ Other

How long is the degree in years? (if it varies across programs, please provide the range)

How many training positions/slots are maximally available per year in your country? (If your training programs enroll only every

2 years, for example, please divide in half?)

Please select all the parts of the training process where genetic counselors are involved

- ☐ *In teaching curriculum
- ☐ *In supervising local rotations
- ☐ *In supervising rotations that your own students complete in other countries
- ☐ *In supervising research/thesis
- ☐ *In other ways? (please comment)

Please add any additional comments to help us understand how genetic counselor training occurs in your country.

If you do not have genetic counseling training in your country, please describe where/how were were genetic counselors trained?

How are genetic counselors regulated in your country (please check all that apply)

- ☐ There is a professional organization credential (certification, registration, eg. ABGC, CAGC, EBMG, HGSA)
- ☐ There is a national form of recognition or regulation (e.g a law that regulates GC practice, such as through a Health Care Profession Councils, like HPCSA HCPC)
- ☐ There is a state or provincial regulation process in at least some parts of the country (sometimes called licensure or registration).
- ☐ There is not currently any mechanism for regulation (feel free to comment here if in process)
- ☐ Other

Please provide the URL for any relevant professional regulation websites

For a person newly applying for a credentialing/regulation what are the requirements? (check all that apply)

- ☐ Written Exam
- ☐ Oral Exam or Practical assessment (observed counseling session)
- ☐ Supervision and/or Case recording

- ☐ Case Studies and Essays
- ☐ Case Logbook
- ☐ Additional supervised clinical training after graduation (list years required)
- ☐ Continuing Education documentation
- ☐ ____ Other or explanatory comments (please describe)
- ☐ Not applicable, there is no credentialling or regulation in my country.

Please add any comments to help us further understand the professional regulation of genetic counselors in your country.

Now we want to ask you some questions about the status of genetic counseling in your country, with an emphasis on what has changed in the past 5 years. Please write short answers (no more than 50 words to each) to the following questions, and if there are any published articles or websites that we can reference to document the answers, please add them. If you are not aware of changes but suggest we reach out to someone else for details, please include a name and email address.

What has changed in the past 5 years in regards to: Curriculum approaches

What has changed in the past 5 years in regards to: Clinical training approaches

What has changed in the past 5 years in regards to: Research in genetic counseling

What has changed in the past 5 years in regards to: Numbers of genetic counselors in practice

What has changed in the past 5 years in regards to: Registration/regulation

What has changed in the past 5 years in regards to: Roles of genetic counselors (e.g. clinical, including different specialities, research, laboratories, industry, teaching)

What has changed in the past 5 years in regards to: Service delivery models - for example, who do you practice with (geneticists vs. mainstreaming?)

Please describe the way other non-genetics providers are involved and what changes may have occurred in the past 5 years (for example, non-genetics providers are increasingly ordering tests, obtaining consent for services, hiring GCs, etc).

What has changed in the past 5 years in regards to: Reimbursement for genetic counseling services

What has changed in the past 5 years in regards to: Other areas not listed above?

Which of these do you see as the most important/impactful change? Why?

Currently, what are the biggest opportunities for genetic counselors in your country?

Currently, what are the biggest challenges for genetic counselors in your country?

How has telehealth/COVID-19 changed the practice of genetic

counseling in your country?

What are the expectations/hopes for genetic counseling as a profession in the next 5 years?

Block 1

If we need to reach you to clarify any of the responses in this survey, please provide an email address.

If you would like to be personally acknowledged in a publication for your help in completing this survey, please list your name (and/or those who helped you complete the survey) here. Doing so implies permission to be listed in an acknowledgement section of a paper.

If you consulted with colleagues within your country when completing this survey, please list their names and roles here, for our background information. (For example, "I consulted with the president of X organization and Y organization to obtain ... information".)

Are there other countries in your region that are planning to have either genetic counseling practice or training that you can give us some information about? If you know someone we can talk to for more information please also include their email.

Powered by Qualtrics
